# A New Measurement Method for BDS Inter-Satellite Link Based on Co-Frequency Co-Time Full Duplex System

**DOI:** 10.3390/s25113538

**Published:** 2025-06-04

**Authors:** Hao Feng, Zhuo Yang, Hong Ma, Yiwen Jiao, Tao Wu, Hongbin Ma, Qimin Chen

**Affiliations:** 1 Graduate School, Space Engineering University, Beijing 101416, China; fenghao123@hgd.edu.cn (H.F.); yangzhuo0513@163.com (Z.Y.); cqm292979@163.com (Q.C.); 2 Key Laboratory of Intelligent Space TTC&O, Ministry of Education, Beijing 101416, China; jiaoyiwen1985@163.com (Y.J.); wt_ttco2023@163.com (T.W.); hongbin_ma@163.com (H.M.)

**Keywords:** inter-satellite link, Co-frequency Co-time Full Duplex (CCFD), two-way measurement, BeiDou-3, ranging accuracy

## Abstract

To meet the urgent need for high-precision ranging and large-capacity transmission in the current BeiDou-3 inter-satellite link system, this paper proposes a novel two-way measurement method based on Co-frequency Co-time Full Duplex (CCFD) system. This approach effectively addresses the limitations of traditional Time-Division Half-Duplex (TDHD) systems, such as complex link establishment processes, constrained ranging accuracy, and limited transmission efficiency. Based on the spatial configuration of the BeiDou-3 satellite navigation constellation, a dynamic link constraint model is constructed, and a comprehensive link budget analysis is conducted for the entire inter-satellite measurement process. The fundamental principle, system model, and key errors of the two-way measurement in CCFD are derived in detail. Theoretical analysis and experimental simulations demonstrate that the proposed CCFD system is feasible and achieves remarkable ranging accuracy improvements. At a carrier-to-noise ratio of 61.6 dBHz, the system attains 1σ ranging accuracy of 1.9 cm, representing a 51.3% enhancement over the 3.9 cm accuracy of the TDHD system. When operating at 69.3 dBHz, the precision further improves to 0.8 cm, outperforming TDHD’s 2.2 cm by 66.8%. The introduction of CCFD technology can significantly enhance the performance level of the BeiDou-3 satellite navigation system, demonstrating broad application prospects for the future.

## 1. Introduction

The inter-satellite link (ISL), as an integrated dynamic network between satellites and between satellites and ground stations, combines measurement and data transmission functions, bringing significant changes to the traditional operation mode of the Global Navigation Satellite System (GNSS) [1,2]. The primary functions of navigation ISLs can be summarized as follows: (1) achieving precise inter-satellite ranging, improving the geometric configuration of the network, and enhancing the accuracy of orbit determination and time synchronization; (2) utilizing real-time inter-satellite data transmission to increase the update frequency of navigation broadcast ephemeris; (3) enabling autonomous inter-satellite navigation in the absence of ground station support, enhancing the system’s survivability and wartime operational capabilities; (4) addressing the limitations of regional tracking station monitoring capabilities, ensuring global service performance; and (5) real-time monitoring of the integrity of navigation information, improving the system’s availability and reliability [3,4,5]. Currently, major spacefaring nations are accelerating the construction and refinement of ISL systems, striving to provide higher-quality navigation service performance on a global scale [6].

The development of the Global Positioning System (GPS) inter-satellite links began early and has reached the highest level of maturity. As early as the 1980s, the United States initiated research on ISL for the GPS, driven initially by the need for real-time monitoring of global nuclear tests and to enhance the survivability of GPS during wartime [7]. In 1984, Ananda, M. P. et al. first proposed the fundamental framework for autonomous navigation, providing theoretical analysis results that were subsequently adopted by the U.S. military [2]. By 1990, the theoretical design and data simulation for the GPS Block IIR ISL were completed, demonstrating that, without ground support, the user range error (URE) could be maintained below 6 m for up to 180 days [7]. In 1997, the first GPS Block IIR satellite equipped with ISL payloads was developed, tested, and launched. Rajan et al. conducted orbit determination experiments using inter-satellite ranging data from four GPS Block IIR satellites, with results showing that the URE could be better than 3 m over 75 days [8]. To date, GPS has launched four types of satellites: GPS IIR, GPS IIR-M, GPS IIF, and GPS III, with their corresponding operational characteristics summarized in Table 1 [9]. GPS satellites utilize UHF-band wide-beam antennas to establish ISLs, employing a polling-based time-division ranging system, where only one satellite transmits at any given time while the others receive [10]. Currently, the United States is developing third-generation navigation satellites, divided into three phases: GPS IIIA, GPS IIIB, and GPS IIIC. Although GPS III was originally planned to use Ka-band (22.55–23.55 GHz) and V-band (59.3–64 GHz) frequencies, the currently launched satellites of GPS III still operate in the UHF band due to various reasons [11]. Additionally, ongoing projects are exploring the integration of laser payloads into future GPS satellites [12].

Facing intense competition in the global navigation arena and challenges in establishing foreign ground stations, Russia has made significant investments in developing inter-satellite link (ISL) technologies for its GLONASS system. The current implementations primarily serve GLONASS-M and GLONASS-K satellites, each demonstrating distinct technological approaches. The GLONASS-M series satellites utilize S-band wide-beam antennas to establish ISLs and employ a simplex time-division mode to measure inter-satellite pseudo-range values, achieving systematic and random errors of 0.3 m and 0.4 m, respectively [13]. The GLONASS-K ISLs adopt a pulsed laser system, enabling centimeter-level inter-satellite ranging accuracy and medium data transmission rates (50 kbit/s), with the fastest link establishment time being less than 10 s [14]. Looking forward, Russia’s next-generation GLONASS-K2 satellites will feature enhanced capabilities through two-phased array antennas transmitting FDMA/CDMA signals. This upgrade integrates both laser and radio frequency ISL technologies while incorporating higher-precision atomic clocks, promising substantial improvements in system performance and navigation accuracy [15].

The Galileo navigation constellation currently does not employ ISLs, but the European Space Agency (ESA) is actively conducting exploratory research on ISL technology. In the realm of radio frequency ISLs, ESA has initiated two projects: GNSS+ and ADVISE. In the GNSS+ project demonstration, the ISLs operate in the C-band, utilizing a time-division multiple access (TDMA) ranging and communication system. This system enables bidirectional dual-frequency pseudo-range measurements between satellites, achieving an accuracy of 1–2 cm [16]. Building on this, ESA proposed the ADVISE project, which significantly reduced payload mass and radio frequency power consumption by improving ISL design and algorithms. This solution employed a frequency-hopping time-division multiple access (FTDMA) system, achieving orbit determination accuracy of approximately 1 cm and time synchronization performance better than 0.4 ns [17]. Simultaneously, ESA is actively exploring optical quantum links (OQLs) and laser links (Kepler), aiming to investigate new directions for ISL development and enhance the performance of navigation systems [18].

**Figure 1:**
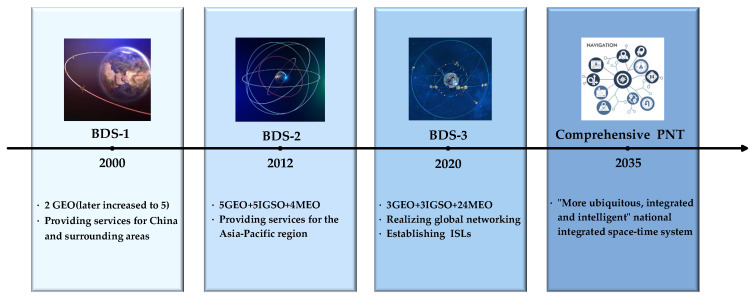
The development timeline of the BeiDou satellite navigation system.

Since the completion of the BeiDou-1 dual-satellite system at the end of 2000, China has adopted a “three-step” development strategy and officially completed the construction of the BeiDou-3 Global Navigation Satellite System (BDS-3) on 31 July 2020 [19]. Furthermore, China plans to establish a “more ubiquitous, integrated, and intelligent” national comprehensive Positioning, Navigation, and Timing (PNT) system by around 2035 [20,21]. Figure 1 illustrates the development timeline of the BeiDou satellite navigation system. Between 2015 and 2016, China launched five experimental satellites (I1-S, I2-S, M1-S, M2-S, and M3-S), equipped with Ka-band ISL payloads. These satellites conducted in-orbit tests on technologies such as topology structure, communication protocols, antenna design, and autonomous navigation algorithms, achieving orbit determination accuracy better than 2 m [22]. Starting in 2017, BDS-3 satellites began carrying ISL payloads to enable inter-satellite measurement and communication. By December 2018, the initial BDS-3 constellation was successfully established, consisting of 18 Medium Earth Orbit (MEO) satellites and 1 Geostationary Earth Orbit (GEO) satellite [23]. Guo et al. processed 30 days of ISL measurement data from 18 MEO satellites using a distributed approach based on the extended Kalman filter, demonstrating that the three-dimensional orbital error could be less than 2.30 m over 30 days [24]. Xie et al. analyzed the orbit determination performance of 18 MEO satellites and 1 GEO satellite using 43 days of ISL measurement data from 2019. The results showed that the radial orbit error of the MEO satellites was on the order of 2–4 cm, while that of the GEO satellite was on the order of 8–10 cm [25].

Currently, all BDS-3 satellites are equipped with Ka-band ISL payloads, enabling precise orbit determination and time synchronization through inter-satellite precision measurement and data transmission. Zhang et al. analyzed the inter-satellite ranging performance using 30 days of measurement data from the complete BDS-3 constellation, revealing that the ISL ranging accuracy was better than 5 cm [26]. Guo et al. conducted an experimental analysis using one month of raw observation data from 29 BDS-3 satellites, consistently demonstrating that the time synchronization accuracy of each satellite was better than 0.4 ns [6]. The BeiDou ISL communication and measurement system employs a time-division half-duplex mode, establishing narrow-beam directional links based on a phased-array mechanism. Each satellite, following a time-slot schedule uploaded from the ground, establishes links with other satellites (or ground anchor stations) in a polling manner. The BDS-3 ISL system divides the timeline into superframes (1 min), with each superframe further subdivided into 20 time slots (3 s). Within a single time slot, the linked satellites alternately transmit signals to each other during their respective 1.5 s intervals, completing two-way inter-satellite measurement and communication [26,27,28].

Currently, ISLs in major global navigation systems predominantly employ a time-division half-duplex (TDHD) mode. While numerous scholars have focused on analyzing link performance based on raw inter-satellite observation data, such as orbit determination accuracy, time synchronization precision, and routing transmission efficiency, research on the operational mechanisms of ISLs remains limited. The TDHD mode, which prevents simultaneous transmission and reception on the same frequency, results in unidirectional transmission rates being only 50% of the total channel capacity. Additionally, the link establishment process is complex, and timestamp calculation and error correction are prone to significant inaccuracies. These limitations lead to issues such as low communication efficiency, high latency, and restricted inter-satellite measurement accuracy [3]. In contrast, Co-frequency Co-time Full Duplex (CCFD) technology, a key innovation proposed during the 5G era, has matured significantly in recent years [29,30,31]. Compared to traditional time-division and frequency-division systems, CCFD technology enables wireless devices to achieve simultaneous signal transmission and reception on the same frequency. Relative to time-division systems, CCFD technology allows concurrent signal transmission and reception, theoretically doubling the information throughput within the same time interval. In contrast to frequency-division systems, CCFD technology can effectively conserve bandwidth resources, theoretically improving spectral efficiency by a factor of two. Currently, numerous researchers have begun dedicating efforts to the study of CCFD technology, covering a wide range of fields such as massive multiple-input multiple-output systems, low-power wide-area networks, relay communication networks, Wi-Fi systems, Long-Term Evolution systems, and cognitive radio [29]. Introducing CCFD into ISL measurement and communication processes could effectively overcome the constraints of TDHD systems, which are unable to simultaneously transmit and receive signals on the same frequency. Moreover, it would simplify the link establishment process and enable continuous inter-satellite transmission and precise measurement. In terms of transmission, CCFD could double the inter-satellite transmission rate, measurement frequency, and data update rate. For measurement, CCFD could effectively mitigate ranging errors caused by relative motion, clock bias, and Doppler effects between satellites. This would eliminate the need for complex models to improve ranging accuracy and enhance the performance of inter-satellite measurements.

In this paper, we propose a novel inter-satellite two-way measurement method based on the CCFD system to enhance the overall performance of the ISL network. Section 2 analyzes the self-interference suppression performance of the CCFD system from three domains and presents the current total self-interference suppression level. Section 3 provides a detailed analysis of the spatial configuration of the BDS-3 satellite navigation constellation, constructing a dynamic link constraint model based on satellite visibility and antenna directivity. Section 4 conducts a comprehensive link budget analysis of the BDS-3 ISL. Section 5 systematically introduces the proposed two-way measurement method for ISL, including the fundamental principle, system model, and key errors, theoretically evaluating the system performance under the new framework. Section 6 presents experimental simulations and analyses, autonomously constructing a BDS-3 ISL scenario and a digital simulation verification platform to capture inter-satellite topological characteristics, validate the feasibility of the full-duplex system, and analyze pseudo-code ranging performance under the new framework. Section 7 analyzes the experimental results and discusses potential directions for future research. Section 8 provides a comprehensive summary and synthesis of the research findings presented in this paper.

## 2. CCFD Self-Interference Suppression Performance

**Figure 2:**
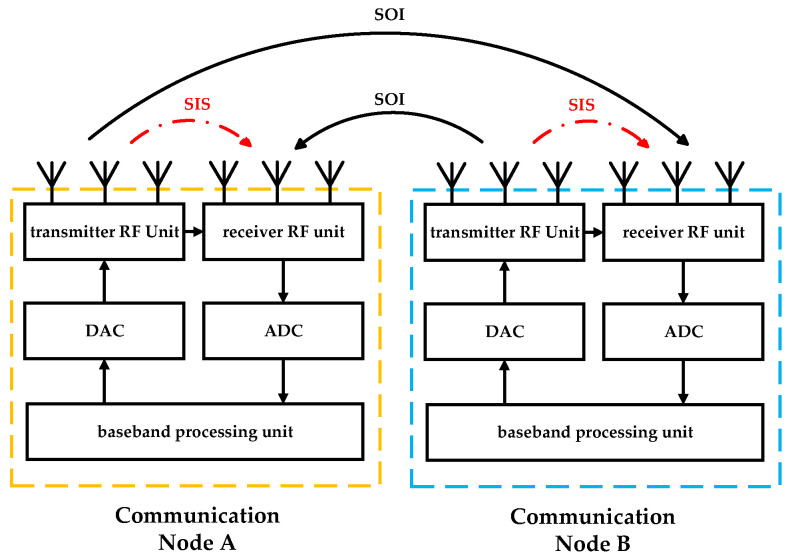
Basic system model of Co-time Co-frequency Full Duplex (CCFD).

The basic system model of CCFD is illustrated in Figure 2. During the reception of the Signal of Interest (SOI), due to the long distance between the transmitter and receiver, the signal experiences path loss during spatial transmission, resulting in low received signal power and high demands on receiver sensitivity. In contrast, for the Self-Interference Signal (SIS), since the transmitter and receiver are located at the same communication node and are spatially very close, the received signal power is at least 100 dB higher than the SOI [32]. This significantly degrades the quality of the SOI reception.

In the CCFD communication mode, although it is possible to transmit and receive signals simultaneously at the same frequency, the target signal is affected by self-interference from the local high-power transmitted signal. This interference prevents the communication system from effectively extracting the valuable signal. Therefore, effectively suppressing system self-interference is a key technical challenge that must be addressed to realize the CCFD framework [33].

**Figure 3:**
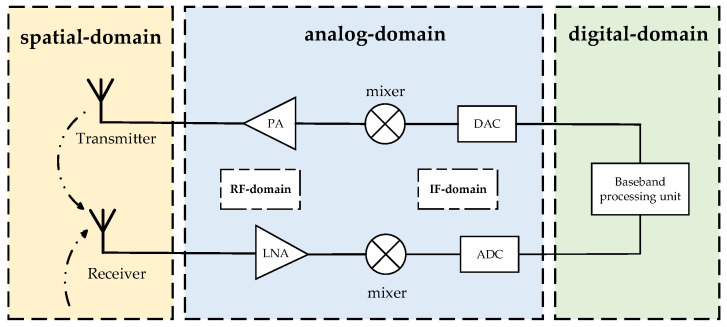
Schematic diagram of self-interference suppression locations in CCFD systems.

Currently, extensive research is being conducted on CCFD self-interference suppression techniques. Based on the location of self-interference suppression, these techniques can be broadly categorized into spatial-domain (antenna-domain) self-interference suppression, analog-domain self-interference suppression, and digital-domain self-interference suppression [34]. Analog-domain self-interference suppression can be further divided into radio frequency (RF) and intermediate frequency (IF) self-interference suppression, with RF-domain suppression being the primary focus. A detailed schematic diagram is provided in Figure 3.

### 2.1. Spatial-Domain Self-Interference Suppression

Spatial-domain self-interference suppression primarily aims to reduce the near-field coupling effect between antennas by improving the spatial isolation between transmitting and receiving antennas. It can be divided into two main categories: passive suppression and active suppression. Passive suppression methods mainly include antenna separation [35], orthogonal polarization [36], near-field cancellation [37], and decoupling structure design [38]. Active suppression techniques mainly rely on transmit–receive beamforming [39].

Since BDS-3 ISLs utilize a Ka-band phased-array system, the focus of spatial-domain self-interference suppression is placed on array antennas operating within the central frequency range of 20 GHz to 30 GHz. As shown in Table 2, the current self-interference suppression performance of array antennas in the spatial domain can achieve a maximum level exceeding 100 dB. This significantly reduces the power of self-interference signals at the antenna, providing a strong foundation for further suppression in subsequent stages.

### 2.2. RF-Domain Self-Interference Suppression

Spatial-domain self-interference suppression has certain limitations and cannot eliminate SIS. The residual SIS remains stronger than the desired signal, necessitating further suppression in the RF domain [48]. RF-domain self-interference suppression involves processing at the RF front-end, where the idea is to estimate the residual SIS and remove it from the received RF signal, thereby preventing saturation of nonlinear components in the RF receiver chain [49].

Based on current research, RF-domain self-interference suppression techniques can be broadly categorized into three types: direct RF coupling interference suppression [50], digitally assisted RF interference suppression [33], and high-isolation device design. Table 3 summarizes the status of RF-domain self-interference suppression. For received signals within the central frequency range of 20 GHz to 30 GHz, current techniques such as multi-tap channel reconstruction and the development of high-isolation devices can achieve RF-domain self-interference suppression exceeding 35 dB.

### 2.3. Digital-Domain Self-Interference Suppression

After suppression in the RF domain, the residual self-interference signal needs to be further suppressed in the digital domain to enable the identification and demodulation of the desired signal. In the CCFD system, the near-end transmitted signal is considered known, while the characteristics of the self-interference channel are unknown. Therefore, digital-domain self-interference suppression requires the use of the known transmitted self-interference signal to estimate and compensate for the self-interference channel, achieving the goal of self-interference suppression [55].

Current research on digital-domain self-interference suppression techniques primarily includes self-interference channel reconstruction suppression [56], adaptive filtering-based self-interference suppression [57], and nonlinear self-interference suppression [58]. Table 4 presents the status of digital-domain self-interference suppression. With the introduction of advanced technologies such as artificial intelligence and deep learning, the best suppression performance in the digital domain can exceed 45 dB.

Yu et al. conducted a study on a spread-spectrum telemetry system with a 20 MHz bandwidth, utilizing multi-tap RF cancellation and digital nonlinear self-interference suppression methods. Through simulations and experimental validation, they achieved self-interference suppression levels of 41.5 dB in the RF domain and 38 dB in the digital domain, resulting in a combined suppression of approximately 81 dB [64]. This research provides a significant reference value for the CCFD communication and measurement systems in BDS-3 ISLs. Based on the current research, it is evident that for a CCFD spread-spectrum measurement and communication system using Ka-band array antennas with an RF reception bandwidth of 20 MHz, a total self-interference suppression level of approximately 180 dB can be achieved. This sets a performance benchmark for the feasibility demonstration of CCFD technology in the following discussion.

## 3. BDS-3 Constellation Configuration and ISL Establishment Constraints

The BDS-3 constellation is a multi-layered heterogeneous hybrid system, consisting of 3 GEO satellites, 3 Inclined Geosynchronous Orbit (IGSO) satellites, and 24 MEO satellites. The MEO satellites are arranged in a Walker 24/3/1 constellation configuration, featuring an orbital altitude of 21,528 km and an inclination of 55°. This configuration distributes the 24 MEO satellites across three evenly spaced orbital planes, with eight satellites uniformly positioned within each plane. Additionally, the phase difference between corresponding satellites in adjacent orbital planes is 15°. For the 24 MEO satellites configured in the Walker constellation, the satellites within the three orbital planes are labeled as MEO_1m_, MEO_2n_, and MEO_3k_, where m, n, k = 1, 2, …, 8. The 3 IGSO satellites have an inclination angle of 55° and a theoretical orbital altitude of 35,786 km. The 3 GEO satellites are positioned at longitudes of 80° E, 110.5° E, and 140° E, with a theoretical orbital altitude of 35,786 km.

The BDS-3 constellation features a large number of orbital planes and complex orbital types, and the relative positions and spatial relationships between satellites change rapidly over time. Therefore, the following constraints must be considered when establishing ISLs.

### 3.1. Satellite Visibility Constraints

The fundamental condition for establishing a link between two satellites is that they are mutually visible in space, meaning there is no Earth obstruction between them. Additionally, to avoid interference and effects from the troposphere and ionosphere on the ISL signals, the visibility constraint can be strengthened by requiring that the inter-satellite communication zone be at least 1000 km above the Earth’s surface.

**Figure 4:**
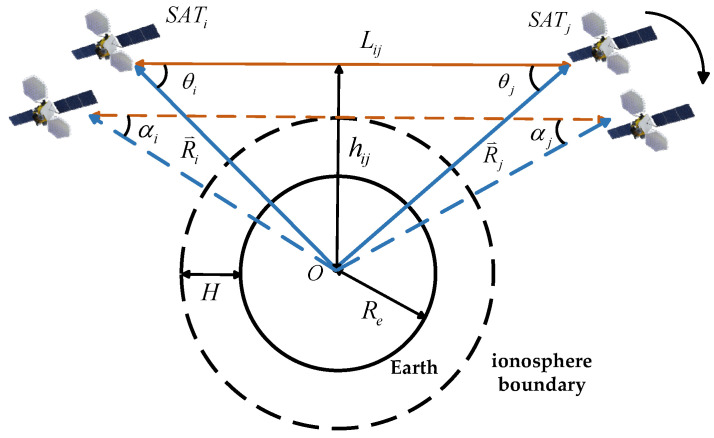
Schematic diagram of satellite visibility constraints.

As shown in Figure 4, *SAT_i_* and *SAT_j_* represent the two satellites establishing the link, *O* denotes the center of the Earth, $R_{e}$ is the Earth’s radius, and H is the height of the ionosphere. The vectors ${\vec{R}}_{i}$ and ${\vec{R}}_{j}$ represent the direction vectors from the Earth’s center to satellites *i* and *j*, respectively. $L_{ij}$ is the distance between the two satellites, $h_{ij}$ is the distance from the Earth’s center to the line connecting satellites *i* and *j*, $\theta_{i}$ is the angle between the line connecting satellite *i* to satellite *j* and the line connecting satellite *i* to the Earth’s center, and $\theta_{j}$ is the angle between the line connecting satellite *j* to satellite *i* and the line connecting satellite *j* to the Earth’s center. $\alpha_{i}$ and $\alpha_{j}$ are Earth occlusion angles of satellites *i* and *j*.

To ensure unobstructed inter-satellite signal transmission and guaranteed communication quality, the spatial positional relationship between satellite *i* and satellite *j* must satisfy the following equation:

(1) $$h_{ij}\geq R_{e}+H$$

Using ${\vec{R}}_{i}$ and ${\vec{R}}_{j}$ to represent $h_{ij}$, the distance constraint formula for inter-satellite visibility can be derived as follows:

(2) $$\frac{\left|{\vec{R}}_{i}\times {\vec{R}}_{i}\right|}{L_{ij}}=\frac{\left|{\vec{R}}_{i}\times {\vec{R}}_{j}\right|}{\left|{\vec{R}}_{i}-{\vec{R}}_{j}\right|}\geq R_{e}+H$$

When the line connecting satellite *i* and satellite *j* is tangent to the ionosphere boundary, the angle between the satellite connection line and the line from the satellite to the Earth’s center can be calculated, resulting in the angle constraint formula for inter-satellite visibility:

(3) $$\left\{\begin{array}{l} \theta_{i}\geq \alpha_{i}=\arcsin\frac{R_{e}+H}{\left|{\vec{R}}_{i}\right|}\\ \theta_{j}\geq \alpha_{j}=\arcsin\frac{R_{e}+H}{\left|{\vec{R}}_{j}\right|} \end{array}\right.$$

### 3.2. Antenna Directivity Constraints

The BDS-3 employs phased-array antennas for its ISLs. By controlling the phase relationships of the antenna elements, the system can achieve beam scanning over a wide spatial range, enabling effective signal transmission. The BDS-3 ISLs operate in the Ka-band, which features relatively narrow antenna beams. By adjusting the beam direction, the system can establish point-to-point signal transmission links. Furthermore, phased-array antennas have a maximum beam scanning angle. Therefore, when establishing ISLs, the antenna directivity constraints must be considered. Specifically, the satellite pair planning to establish a link must simultaneously fall within each other’s beam scanning range to ensure effective link establishment.

**Figure 5:**
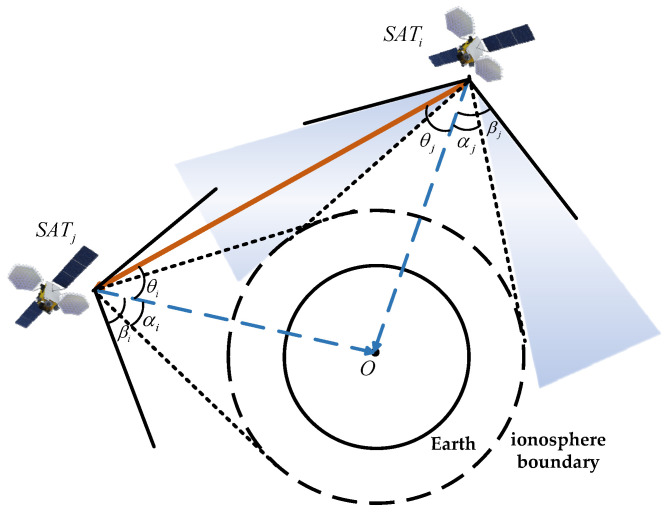
Schematic diagram of antenna directivity constraint.

Figure 5 illustrates the antenna directivity constraints, where $\beta_{i}$ and $\beta_{j}$ represent the maximum beam scanning ranges of satellite *i* and satellite *j*, respectively. If both satellites are within each other’s visibility range, the following antenna directivity constraint conditions must be satisfied, as expressed in the equation below:

(4) $$\left\{\begin{array}{l} \alpha_{i}\leq \theta_{i}\leq \beta_{i}\\ \alpha_{j}\leq \theta_{j}\leq \beta_{j} \end{array}\right.$$

Considering practical engineering constraints, the maximum beam scanning range for BDS-3 can be set to 60° [25]. Taking into account both satellite visibility and antenna directivity constraints, a theoretical analysis of the ISL antenna scanning range and the maximum communication distance can be conducted. For MEO-MEO type links and MEO-GEO/IGSO type links, the maximum communication distance can be calculated as follows:

(5) $$L_{\max(MEO-MEO)}=2\sqrt{{\left(R_{e}+H+H_{M}\right)}^{2}-{\left(R_{e}+H\right)}^{2}}$$

(6) $$L_{\max(MEO-GEO/IGSO)}=\sqrt{{\left(R_{e}+H+H_{M}\right)}^{2}-{\left(R_{e}+H\right)}^{2}}+\sqrt{{\left(R_{e}+H+H_{G}\right)}^{2}-{\left(R_{e}+H\right)}^{2}}$$

where $L_{\max(MEO-MEO)}$ represents the maximum inter-satellite distance for MEO-MEO type links, and $L_{\max(MEO-GEO/IGSO)}$ represents the maximum inter-satellite distance for MEO-GEO/IGSO type links. $H_{M}$ denotes the theoretical orbital altitude of MEO satellites, and $H_{G}$ denotes the theoretical orbital altitude of GEO/IGSO satellites. The Earth occlusion angles for MEO satellites and GEO/IGSO satellites can be calculated using the following equations:

(7) $$\alpha_{MEO}=\arcsin\frac{R_{e}+H}{R_{e}+H+H_{M}}$$

(8) $$\alpha_{GEO/IGSO}=\arcsin\frac{R_{e}+H}{R_{e}+H+H_{G}}$$

where $\alpha_{MEO}$ represents the Earth occlusion angle for MEO satellites, and $\alpha_{GEO/IGSO}$ represents the Earth occlusion angle for GEO/IGSO satellites. Taking $R_{e}$ = 6378 km, H = 1000 km, $H_{M}$ = 21,528 km, and $H_{G}$ = 35,786 km, and substituting these values into Equations (5)~(8), the maximum inter-satellite communication distance and the Earth occlusion angle can be calculated. The calculation results are shown in Table 5.

Simulation results indicate that the maximum inter-satellite communication distance for MEO-MEO type links can reach 55,897.12 km, while for MEO-GEO/IGSO type links, it can reach 70,477.33 km. The Earth occlusion angles for MEO satellites and GEO/IGSO satellites are 14.79° and 9.84°, respectively. In the next step of inter-satellite topology analysis, inter-satellite visibility and antenna directivity constraints should be considered. To ensure signal transmission quality with a sufficient margin, the beam scanning range for MEO satellites can be set to [15°, 60°], while for GEO/IGSO satellites, it can be set to [10°, 60°].

## 4. Comprehensive Link Budget Analysis of the BDS-3 ISL

Various link losses should be calculated to quantitatively assess the signal transmission quality based on the analysis of inter-satellite link topology characteristics. The current link performance is compared with system requirements such as data transmission rate, bit error rate, and ranging accuracy. Through iterative adjustments of signal transmission power and antenna gain, the ISL design is refined to meet the specified performance metrics required by the satellite navigation system.

### 4.1. Analysis of Inter-Satellite Link Loss

During the propagation of ISL signals, the signal power does not remain constant but decreases due to various losses, including free-space transmission loss, feeder loss, polarization loss, and pointing loss. Among these, free-space transmission loss is the predominant factor.

Free-space transmission loss refers to the signal attenuation caused by the propagation of radio signals in free space. The corresponding formula for the BDS-3 ISL signal can be expressed as follows:

(9) $$L_{BDS\_ISL}={\left(\frac{4\pi d_{BDS\_ISL}}{\lambda_{BDS\_ISL}}\right)}^{2}={\left(\frac{4\pi d_{BDS\_ISL}f_{BDS\_ISL}}{c}\right)}^{2}$$

where $L_{BDS\_ISL}$ is the free-space transmission loss of the BDS-3 ISL signal, $d_{BDS\_ISL}$ is the geometric propagation distance between satellites in m, $f_{BDS\_ISL}$ is the ISL signal carrier frequency in Hz, $\lambda_{BDS\_ISL}$ is the ISL signal carrier wavelength in m, and c is the speed of light, with a value of $3\times 10^{8}$ m/s. Then the received signal power of satellites can be expressed as follows:

(10) $$P_{r\_BDS}=\frac{P_{t\_BDS}G_{t\_BDS}G_{r\_BDS}}{L_{BDS\_ISL}}$$

where $P_{r\_BDS}$ is the received signal power of satellites in W, $P_{t\_BDS}$ is the transmission signal power of satellites in W, $G_{t\_BDS}$ and $G_{r\_BDS}$ are the transmitting antenna gain and the receiving antenna gain in dBi, respectively.

Typically, we use the Equivalent Isotropic Radiated Power (EIRP) as an important metric to evaluate the transmission capability of a satellite. It can be expressed in dB as follows:

(11) $$\left[\mathrm{EIRP}\right]=[P_{t\_BDS}]+[G_{t\_BDS}]-[L_{S\_BDS}]$$

where $L_{S\_BDS}$ is the transmission circuit feed loss in dB. To better characterize the signal reception and amplification capability of the satellite receiving system, we typically use the receiver antenna quality factor for evaluation, which can be expressed in dB as follows:

(12) $$\left[G/T]=[G_{r\_BDS}]-[T_{s}\right]$$

where G/T is the receiver antenna quality factor in dB/K, and $T_{s}$ is the total noise temperature of the receiving system in K.

During the transmission of ISLs, in addition to free-space transmission loss, there are a series of other losses, including receiver feeder loss, antenna polarization loss, and antenna pointing loss. Taking into account various losses and to better reflect the performance of the satellite transmission and reception systems, Equation (10) can be rewritten in dB form as follows:

(13) $$\left[P_{r\_BDS}]=[\mathrm{EIRP}]+[G/T]+[T_{s}]-[L_{BDS\_ISL}]-[L_{N\_BDS}]-[L_{P\_BDS}]-[L_{D\_BDS}]-[L_{M}\right]$$

where $L_{N\_BDS}$ is the receiver feeder loss in dB, $L_{P\_BDS}$ is the antenna polarization loss in dB, $L_{D\_BDS}$ is the antenna pointing loss in dB, and $L_{M}$ is the link margin of the system in dB.

### 4.2. Analysis of Inter-Satellite Link Signal Quality

During the inter-satellite measurement and communication process, it is necessary to analyze the signal transmission quality to determine whether it meets various system performance requirements. Signal quality is typically measured using the signal-to-noise ratio (SNR), but the value of SNR depends on the noise bandwidth of the receiver, which can complicate its application. To facilitate performance comparisons between different receivers, the carrier-to-noise ratio can be used as an evaluation metric, and its equation can be expressed as follows:

(14) $$C/N_{0}=\frac{P_{r\_BDS}}{N_{0}}$$

where $C/N_{0}$ is the carrier-to-noise ratio of the received signal in dBHz, and $N_{0}$ is the noise power spectral density in dBW/Hz. The equation of $N_{0}$ can be expressed as follows:

(15) $$N_{0}=N/B_{f}=kT_{s}$$

where N is the noise power of the receiver in dBW, $B_{f}$ is the received bandwidth of the signal in Hz, and k is Boltzmann’s constant, with a value of $1.38\times 10^{-23}$ J/K. The noise temperature of the receiving system primarily consists of three components: the noise temperature from external sources, the noise temperature generated by cable losses, and the noise temperature produced by the series components in the system. According to Friis’ formula, the total noise temperature of the receiving system can be calculated as follows:

(16) $$T_{s}=T_{a}+(L_{N\_BDS}-1)T_{0}+(F-1)L_{N\_BDS}T_{0}$$

where $T_{a}$ is the noise temperature from external sources in K, F is the noise figure in dB, and $T_{0}$ is the ambient temperature in K. Considering various losses, Equation (14) can be expressed in dB form as follows:

(17) $$\left[C/N_{0}]=[\mathrm{EIRP}]+[G/T]-[L_{BDS\_ISL}]-[L_{P\_BDS}]-[L_{D\_BDS}]-[k]-[L_{M}\right]$$

For inter-satellite communication systems employing BPSK or QPSK digital modulation, a more commonly used evaluation metric is the normalized signal-to-noise ratio, defined as the ratio of energy per bit to noise power spectral density ($E_{b}/N_{0}$). The relationship between $E_{b}/N_{0}$ and $C/N_{0}$ can be expressed as follows:

(18) $$E_{b}/N_{0}=\frac{C/N_{0}}{R_{b\_ISL}}$$

where $R_{b\_ISL}$ is the information transmission rate of the ISL signal in bps.

### 4.3. Analysis of Inter-Satellite Link Margin

For BPSK and QPSK modulation schemes, the following relationships hold:

(19) $$P_{b}=\frac{1}{2}erfc\sqrt{E_{b}/N_{0}}$$

where erfc(·) is the complementary error function, and $P_{b}$ is the system Bit Error Rate (BER).

We can calculate the normalized signal-to-noise ratio based on the system BER requirements. The extent to which the actual normalized signal-to-noise ratio exceeds the required normalized signal-to-noise ratio can be defined as the link margin, which can be expressed as follows:

(20) $$M_{BDS\_ISL}=\frac{{\left(E_{b}/N_{0}\right)}_{real}}{{\left(E_{b}/N_{0}\right)}_{req}}$$

where $M_{BDS\_ISL}$ is the link margin of the BDS-3 ISL, ${\left(E_{b}/N_{0}\right)}_{real}$ is the actual normalized signal-to-noise ratio, and ${\left(E_{b}/N_{0}\right)}_{req}$ is the required normalized signal-to-noise ratio. Combining Equations (17), (18) and (20), the link margin can be expressed in dB as follows:

(21) $$\left[M_{BDS\_ISL}]=[\mathrm{EIRP}]+[G/T]-[L_{BDS\_ISL}]-[L_{P\_BDS}]-[L_{D\_BDS}]-[k]-[R_{b\_ISL}]-[L_{M}]-[{\left(E_{b}/N_{0}\right)}_{req}\right]$$

## 5. Two-Way Measurement Method in CCFD

Introducing CCFD into the BDS-3 ISL system enables simultaneous transmission and reception of signals on the same frequency, thereby enhancing the overall performance of the constellation. This section provides a detailed analysis of the fundamental principle, system model, and key errors of inter-satellite two-way measurement in CCFD. It has been proved in theory that this approach can achieve higher ranging precision.

### 5.1. Principle of Two-Way Measurement Method in CCFD

The two-way measurement method in CCFD is described as follows: (1) In terms of the time slot schedule uploaded from the ground, the satellite pair is identified to establish the link. (2) The two satellites (satellite i and j) agree on the same transmission time and simultaneously send signals to each other. The signals share the same frame structure, with the baseband clock and carrier frequency generated locally by each satellite. (3) While transmitting, each satellite synchronously receives the signal sent by the other satellite. (4) Through spatial-domain, RF-domain, and digital-domain self-interference suppression, the self-interference signal can be reduced to the receiver noise floor, enabling effective signal reception. (5) The satellites execute signal acquisition, tracking, and demodulation processes to recover the information frame, extract the transmission epoch time, and integrate it with the local epoch time to compute the local pseudo-range. (6) The local pseudo-range is embedded into the information frame and transmitted to the other satellite. Both satellites then construct the two-way observation equation using the local pseudo-range and the pseudo-range demodulated from the received information frame. (7) The system performs timestamp calculation and key error correction to derive the modified pseudo-range equation. (8) The system performs summation and difference operations on the modified pseudo-range equation to derive the inter-satellite geometric distance and clock bias, which are necessary for precise orbit determination and time synchronization.

**Figure 6:**
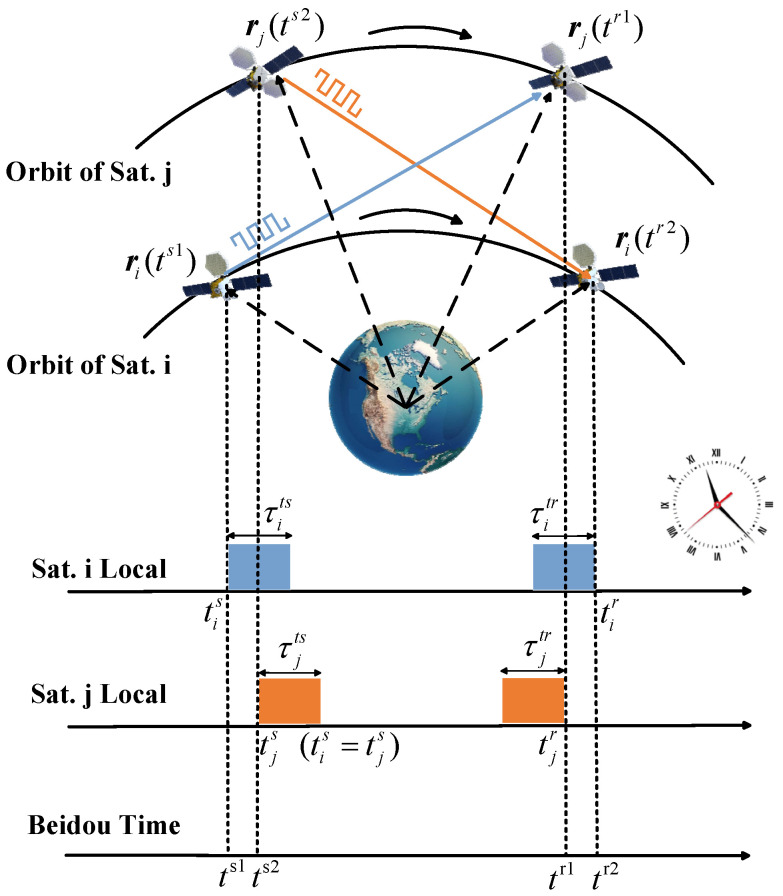
Timing diagram of inter-satellite two-way ranging in CCFD.

Figure 6 illustrates the timing diagram of inter-satellite two-way measurement in CCFD, with the following parameter definitions: (1) $t_{i}^{s}$ is the local transmission time of the satellite i, and $t^{s1}$ is the corresponding BeiDou time (BDT). $t_{j}^{s}$ is the local transmission time of the satellite j, and $t^{s2}$ is the corresponding BDT. (2) $t_{i}^{r}$ is the local reception time of the satellite i, and $t^{r2}$ is the corresponding BDT. $t_{j}^{r}$ is the local transmission time of the satellite j, and $t^{r1}$ is the corresponding BDT. (3) $\tau_{i}^{ts}$ and $\tau_{i}^{tr}$ represent the transmission and reception channel delays of the satellite i, respectively. $\tau_{j}^{ts}$ and $\tau_{j}^{tr}$ represent the satellite’s transmission and reception channel delays j, respectively. (4) $r_{i}(t^{s1})$ and $r_{i}(t^{r2})$ denote the position vectors of the satellite i in the Earth-Centered Inertial (ECI) coordinate system at the transmission and reception times, respectively. $r_{j}(t^{s2})$ and $r_{j}(t^{r1})$ denote the position vectors of the satellite j in the ECI coordinate system at the transmission and reception times, respectively.

### 5.2. System Model of Two-Way Measurement Method in CCFD

Based on the process of inter-satellite two-way measurement, the observation equation can be derived as follows:

(22) $$\begin{array}{l} \rho_{ij}=|r_{j}(t^{r1})-r_{i}(t^{s1})|+c[dt_{j}(t^{r1})-dt_{i}(t^{s1})]+c\delta_{ij}^{sys}+\varepsilon_{ij}\\ \rho_{ji}=|r_{i}(t^{r2})-r_{j}(t^{s2})|+c[dt_{i}(t^{r2})-dt_{j}(t^{s2})]+c\delta_{ji}^{sys}+\varepsilon_{ji} \end{array}$$

where $\rho_{ij}$ and $\rho_{ji}$ represent the locally measured pseudo-ranges of satellites j and i, respectively. $dt_{i}(t^{s1})$ and $dt_{i}(t^{r2})$ are the clock bias between the local time of the satellite i and BDT at times $t^{s1}$ and $t^{r2}$, respectively. $dt_{j}(t^{s2})$ and $dt_{j}(t^{r1})$ are the clock bias between the local time of the satellite j and BDT at times $t^{s2}$ and $t^{r1}$, respectively. The clock bias can be expressed as follows:

(23) $$\begin{array}{l} dt_{i}(t^{s1})=t_{i}^{s}-t^{s1}\\ dt_{i}(t^{r2})=t_{i}^{r}-t^{r2}\\ dt_{j}(t^{s2})=t_{j}^{s}-t^{s2}\\ dt_{j}(t^{r1})=t_{j}^{r}-t^{r1} \end{array}$$

$\delta_{ij}^{sys}$ and $\delta_{ji}^{sys}$ represent the systematic errors of the two-way measurement, while $\varepsilon_{ij}$ and $\varepsilon_{ji}$ denote the ranging noise. The systematic errors can be further expressed as follows:

(24) $$\begin{array}{l} \delta_{ij}^{sys}=\tau_{i}^{ts}+\tau_{j}^{tr}+\tau_{ij}^{rel\_g}+\tau_{ij}^{rel\_p}+\tau_{ij}^{pco}\\ \delta_{ji}^{sys}=\tau_{j}^{ts}+\tau_{i}^{tr}+\tau_{ji}^{rel\_g}+\tau_{ji}^{rel\_p}+\tau_{ji}^{pco} \end{array}$$

where $\tau_{ij}^{rel\_g}$ and $\tau_{ji}^{rel\_g}$ represent the gravitational time delay of the two-way measurement, respectively. $\tau_{ij}^{rel\_p}$ and $\tau_{ji}^{rel\_p}$ denote the time delay of periodic relativistic effects in the two-way measurement, respectively. $\tau_{ij}^{pco}$ and $\tau_{ji}^{pco}$ represent the time delay of phase center offset (PCO) in the two-way measurement, respectively. Therefore, the inter-satellite two-way observation equation can be expressed in detail as follows:

(25) $$\begin{array}{l} \rho_{ij}=|r_{j}(t^{r1})-r_{i}(t^{s1})|+c[dt_{j}(t^{r1})-dt_{i}(t^{s1})]+c(\tau_{i}^{ts}+\tau_{j}^{tr}+\tau_{ij}^{rel\_g}+\tau_{ij}^{rel\_p}+\tau_{ij}^{pco})+\varepsilon_{ij}\\ \rho_{ji}=|r_{i}(t^{r2})-r_{j}(t^{s2})|+c[dt_{i}(t^{r2})-dt_{j}(t^{s2})]+c(\tau_{j}^{ts}+\tau_{i}^{tr}+\tau_{ji}^{rel\_g}+\tau_{ji}^{rel\_p}+\tau_{ji}^{pco})+\varepsilon_{ji} \end{array}$$

Given that the maximum communication distance of BDS-3 ISLs can reach 70,000 km and the relative velocity between satellites can reach several km/s, the effects of signal propagation delay and high-speed satellite motion cannot be overlooked [65]. ISL ranging aims to determine the geometric distance between two satellites at the same instant. Therefore, it is necessary to perform timestamp calculations on the observations to align the inter-satellite pseudo-range measurement to a unified system time. $t^{s1}$ can be selected as the reckoning time ($t_{0}$) in CCFD, and the inter-satellite two-way observation equation can be adjusted as follows:

(26) $$\begin{array}{l} \rho_{ij}=|r_{j}(t_{0})-r_{i}(t_{0})|+\Delta D_{ij}+c\{[dt_{j}(t_{0})+dT_{j}(t^{r1},t_{0})]-[dt_{i}(t_{0})+dT_{i}(t^{s1},t_{0})]\}+c(\tau_{i}^{ts}+\tau_{j}^{tr}+\tau_{ij}^{rel\_g}+\tau_{ij}^{rel\_p}+\tau_{ij}^{pco})+\varepsilon_{ij}\\ \rho_{ji}=|r_{i}(t_{0})-r_{j}(t_{0})|+\Delta D_{ji}+c\{[dt_{i}(t_{0})+dT_{i}(t^{r2},t_{0})]-[dt_{j}(t_{0})+dT_{j}(t^{s2},t_{0})]\}+c(\tau_{j}^{ts}+\tau_{i}^{tr}+\delta_{ji}^{rel\_g}+\delta_{ji}^{rel\_p}+\delta_{ji}^{pco})+\varepsilon_{ji} \end{array}$$

where $\Delta D_{ij}$ and $\Delta D_{ji}$ are the distance corrections for the inter-satellite two-way measurement, respectively. $dT_{i}(t,t_{0})$ is the clock bias correction for satellite i from time t to $t_{0}$, and $dT_{j}(t,t_{0})$ is the clock bias correction for satellite j from time t to $t_{0}$. Comparing Equations (25) and (26), the following equations can be derived as follows:

(27) $$\begin{array}{l} \Delta D_{ij}=|r_{j}(t^{r1})-r_{i}(t^{s1})|-|r_{j}(t_{0})-r_{i}(t_{0})|\\ \Delta D_{ji}=|r_{i}(t^{r2})-r_{j}(t^{s2})|-|r_{i}(t_{0})-r_{j}(t_{0})| \end{array}$$

(28) $$\begin{array}{l} dT_{i}(t^{s1},t_{0})=dt_{i}(t^{s1})-dt_{i}(t_{0})\\ dT_{i}(t^{r2},t_{0})=dt_{i}(t^{r2})-dt_{i}(t_{0})\\ dT_{j}(t^{s2},t_{0})=dt_{j}(t^{s2})-dt_{j}(t_{0})\\ dT_{j}(t^{r1},t_{0})=dt_{j}(t^{r1})-dt_{j}(t_{0}) \end{array}$$

Analyzing the measurement process from satellite i to satellite j, it can be concluded that:

(29) $$|r_{j}(t^{r1})-r_{i}(t^{s1})|=|r_{j}(t^{s1})-r_{i}(t^{s1})|+\int_{0}^{t^{r1}-t^{s1}}v_{j}(t)\cdot e_{ij}(t)dt$$

where $v_{j}(t)$ represents the velocity of satellite j, and $e_{ij}(t)$ denotes the unit direction vector from satellite i to satellite j. Let $f(t)=\int_{0}^{t-t^{s1}}v_{j}(t)\cdot e_{ij}(t)dt$, and perform a first-order Taylor expansion at $t=t^{s1}$, yielding as follows:

(30) $$\begin{array}{l} f(t)=f(t^{s1})+\frac{df}{dt}{|}_{t=t^{s1}}\cdot (t-t^{s1})+R_{1}(t-t^{s1})\\ =v_{j}(t^{s1})\cdot e_{ij}(t^{s1})\cdot (t-t^{s1})+R_{1}(t-t^{s1}) \end{array}$$

where

(31) $$R_{1}(t-t^{s1})=\frac{1}{2}({v^{\prime }}_{j}(\xi )\cdot e_{ij}(\xi )+v_{j}(\xi )\cdot {e^{\prime }}_{ij}(\xi ))\cdot {\left(t-t^{s1}\right)}^{2}$$

where ξ is a value between t and $t^{s1}$. Since the velocity of the satellite changes slowly during a single ranging process and the linked satellites are tens of thousands of kilometers apart, the acceleration of the satellite and the rate of change of the inter-satellite direction can be approximated as zero over a short period, and $R_{1}(t-t^{s1})$ can be neglected. Letting $t=t^{r1}$, Equation (30) can be written as follows:

(32) $$f(t^{r1})=\int_{0}^{t^{r1}-t^{s1}}v_{j}(t)\cdot e_{ij}(t)dt=v_{j}(t^{s1})\cdot e_{ij}(t^{s1})\cdot (t^{r1}-t^{s1})$$

Substituting Equation (32) into Equation (29), it can be concluded that:

(33) $$\left|r_{j}(t^{r1})-r_{i}(t^{s1})|=|r_{j}(t^{s1})-r_{i}(t^{s1})|+v_{j}(t^{s1})\cdot e_{ij}(t^{s1})\cdot (t^{r1}-t^{s1}\right)$$

For the measurement process from satellite j to satellite i, we can similarly derive the following result:

(34) $$\left|r_{i}(t^{r2})-r_{j}(t^{s2})|=|r_{i}(t^{s2})-r_{j}(t^{s2})|+v_{i}(t^{s2})\cdot e_{ji}(t^{s2})\cdot (t^{r2}-t^{s2}\right)$$

where $v_{i}(t)$ represents the velocity of the satellite i, and $e_{ji}(t)$ denotes the unit direction vector from satellite j to satellite i. Substituting Equations (33) and (34) into Equation (27), the following expressions can be obtained as follows:

(35) $$\begin{array}{cc} \Delta D_{ij} & =|r_{j}(t^{s1})-r_{i}(t^{s1})|-|r_{j}(t_{0})-r_{i}(t_{0})|+v_{j}(t^{s1})\cdot e_{ij}(t^{s1})\cdot (t^{r1}-t^{s1})\\ & =v_{j}(t^{s1})\cdot e_{ij}(t^{s1})\cdot (t^{r1}-t^{s1}) \end{array}$$

(36) $$\begin{array}{ll} \Delta D_{ji} & =|r_{i}(t^{s2})-r_{j}(t^{s2})|-|r_{j}(t_{0})-r_{i}(t_{0})|+v_{i}(t^{s2})\cdot e_{ji}(t^{s2})\cdot (t^{r2}-t^{s2})\\ & =|r_{i}(t^{s2})-r_{j}(t^{s2})|-|r_{j}(t^{s1})-r_{i}(t^{s1})|+v_{i}(t^{s2})\cdot e_{ji}(t^{s2})\cdot (t^{r2}-t^{s2}) \end{array}$$

Let $D(t)=|r_{j}(t)-r_{i}(t)|$, representing the spatial distance between satellites at different times. A first-order Taylor expansion of $D(t^{s2})$ at time $t^{s1}$ is performed. Since the duration of a single two-way measurement is short, the inter-satellite acceleration can be approximated as zero [3], allowing the Taylor expansion remainder term to be neglected. The expansion of $D(t^{s2})$ and Equation (36) can be expressed as follows:

(37) $$D(t^{s2})=D(t^{s1})+\frac{dD(t)}{dt}{|}_{t=t^{s1}}(t^{s2}-t^{s1})$$

(38) $$\Delta D_{ji}=\frac{dD(t)}{dt}{|}_{t=t^{s1}}(t^{s2}-t^{s1})+v_{i}(t^{s2})\cdot e_{ji}(t^{s2})\cdot (t^{r2}-t^{s2})$$

To better describe the calculation error, we can define it as follows:

(39) $$\left\{\begin{array}{l} \Delta D_{ji\_spatial\_reckon}=\frac{dD(t)}{dt}{|}_{t=t^{s1}}(t^{s2}-t^{s1})\\ \Delta D_{ji\_d\mathrm{ynamic}\text{\_}\mathrm{compensate}}=v_{i}(t^{s2})\cdot e_{ji}(t^{s2})\cdot (t^{r2}-t^{s2})\\ \Delta D_{ij\_d\mathrm{ynamic}\text{\_}\mathrm{compensate}}=v_{j}(t^{s1})\cdot e_{ij}(t^{s1})\cdot (t^{r1}-t^{s1}) \end{array}\right.$$

where $\Delta D_{ji\_spatial\_reckon}$ is the “spatial reckoning” term resulting from the difference between transmission and reckoning times. $\Delta D_{ij\_d\mathrm{ynamic}\text{\_}\mathrm{compensate}}$ and $\Delta D_{ji\_d\mathrm{ynamic}\text{\_}\mathrm{compensate}}$ are the “dynamic compensation” terms caused by the high-speed motion of the satellites. Therefore, the distance correction term for the inter-satellite two-way measurement can be rewritten as follows:

(40) $$\begin{array}{l} \Delta D_{ij}=\Delta D_{ij\_d\mathrm{ynamic}\text{\_}\mathrm{compensate}}\\ \Delta D_{ji}=\Delta D_{ji\_d\mathrm{ynamic}\text{\_}\mathrm{compensate}}+\Delta D_{ji\_spatial\_reckon} \end{array}$$

Under the monitoring and control of ground stations, the clock bias between BeiDou satellite local time and BDT generally does not exceed 1 ms [3]. Therefore, the clock bias between satellites is typically maintained within 2 ms. The following relationship can be obtained as follows:

(41) $$|t^{s2}-t^{s1}|<2\mathrm{ms}$$

As a navigation satellite, it stores its own broadcast ephemeris and the almanac of other satellites in the constellation. The precision of calculating satellite velocity using the broadcast ephemeris is 0.1 mm/s, while using the almanac yields a precision of dm/s [66]. Therefore, after calculating with the satellite’s local ephemeris and almanac, the correction precision of $\Delta D_{ji\_spatial\_reckon}$ can reach the sub-millimeter level, and the correction error can be considered negligible.

For the TDHD system, the reckoning time is often chosen at the midpoint of the two-way link transmission period. For a 3 s time slot, the difference between the reckoning time and the transmission time is approximately 0.75 s. In this case, the correction precision of the “spatial reckoning” term is only at the centimeter level, which does not meet the high-precision ranging requirements for ISLs. The CCFD system can effectively overcome this issue.

For the “dynamic compensation” term, since the maximum inter-satellite communication distance can reach 70,000 km, the maximum inter-satellite transmission delay is approximately 0.23 s, which means as follows:

(42) $$\begin{array}{l} t^{r1}-t^{s1}\leq 0.23s\\ t^{r2}-t^{s2}\leq 0.23s \end{array}$$

According to Equation (39), the “dynamic compensation” term depends on the receiving satellite’s velocity (which can be calculated from ephemeris data with an accuracy of 0.1 mm/s), the inter-satellite unit direction vector, and the transmission time interval. Since the maximum inter-satellite communication duration is approximately 0.23 s and the inter-satellite distance can extend to tens of thousands of kilometers, the unit direction vector between satellites remains effectively constant during a single transmission slot. Consequently, the “dynamic compensation” term can be corrected to an order of 10^−2^ mm, rendering it negligible in practice.

For the clock bias correction term, since the frequency stability of onboard atomic clocks is generally better than 10^−13^ s/s [25], and the difference between the two-way transmission/reception times and the reckoning time is no more than 0.5 s in CCFD, the maximum value of the clock bias correction term is only on the order of 10^−14^ s, corresponding to a distance on the order of 10^−3^ mm, which is negligible.

In summary, the analysis shows that the error introduced by timestamp reckoning in CCFD is primarily caused by the “dynamic compensation” term in the distance correction. After ephemeris and almanac correction, the precision can reach the sub-millimeter level. Compared to the traditional TDHD system, the correction precision is significantly improved, meeting the requirements for high-precision inter-satellite measurement.

### 5.3. Key Errors of the Two-Way Measurement Method in CCFD

During the process of inter-satellite two-way measurement, various types of errors exist, including channel time delay, gravitational time delay, the time delay of periodic relativistic effects, the time delay of PCO, and ranging noise. The presence of these errors can significantly impact the precision of inter-satellite measurements, necessitating their calculation and compensation.

#### 5.3.1. Channel Time Delay

Channel delay error is one of the significant error terms affecting the precision of inter-satellite measurements. Due to the presence of various nonlinear components in the equipment, along with the effects of aging and temperature drift, the channel delay of the transceiver equipment can vary over time. For a single measurement, since the measurement duration is short and the equipment state changes minimally, the channel delay can be assumed to be constant.

Since the system employs the CCFD framework, allowing simultaneous transmission and reception of signals on the same frequency, an online calibration method can be used to perform real-time calibration of the channel delay. By introducing a self-calibration channel into the ranging equipment, a closed-loop circuit is formed between the transmission and reception channels. Through transmission–reception switching, three different delay combinations are obtained, and the transmission and reception equipment delays can be calculated, achieving a calibration precision better than 0.3 ns [66].

#### 5.3.2. Gravitational Time Delay

Due to the effects of general relativity, gravitational time delay errors occur during signal transmission. These errors can be calculated and corrected using the following formula [6]:

(43) $$\begin{array}{l} \tau_{ij}^{rel\_g}=(1+\gamma )\frac{GM}{c^{2}}\ln(\frac{\left|r_{i}(t^{s1})|+|r_{j}(t^{r1})|+|r_{j}(t^{r1})-r_{i}(t^{s1})\right|}{\left|r_{i}(t^{s1})|+|r_{j}(t^{r1})|-|r_{j}(t^{r1})-r_{i}(t^{s1})\right|})\\ \tau_{ji}^{rel\_g}=(1+\gamma )\frac{GM}{c^{2}}\ln(\frac{\left|r_{j}(t^{s2})|+|r_{i}(t^{r2})|+|r_{i}(t^{r2})-r_{j}(t^{s2})\right|}{\left|r_{j}(t^{s2})|+|r_{i}(t^{r2})|-|r_{i}(t^{r2})-r_{j}(t^{s2})\right|}) \end{array}$$

where γ is the post-Newtonian parameter, and GM is the Earth’s gravitational constant.

#### 5.3.3. Time Delay of Periodic Relativistic Effects

Under the influence of special relativity, satellite clocks exhibit a nominal frequency offset (NFO) and periodic relativistic effects. The NFO can be eliminated by adjusting the clock frequency before transmission, and the correction formula can be expressed as follows [67]:

(44) $$\Delta f_{NFO}=\frac{GM}{c^{2}}(\frac{1}{R_{e}}-\frac{3}{2a})$$

where $\Delta f_{NFO}$ is the NFO in Hz, and a is the satellite orbit semi-long axis in m. The periodic relativistic effects can be calculated and corrected using the following formula [68]:

(45) $$\begin{array}{l} \tau_{ij}^{rel\_p}=\frac{2}{c}(r_{i}(t^{s1})v_{i}(t^{s1})-r_{j}(t^{r1})v_{j}(t^{r1}))\\ \tau_{ji}^{rel\_p}=\frac{2}{c}(r_{j}(t^{s2})v_{j}(t^{s2})-r_{i}(t^{r2})v_{i}(t^{r2})) \end{array}$$

#### 5.3.4. Time Delay of PCO

Inter-satellite measurements are based on the phase center of the transceiver antenna, while the antenna is installed based on its geometric center. During the measurement process, the inconsistency between the antenna’s phase center and its geometric center introduces an error known as the time delay of PCO.

The time delay of PCO can be corrected by the factory default values, which were calibrated by the satellite manufacturer and have been released on the Beidou official website [69]. Alternatively, it can be determined through in-orbit calibration. This method generally utilizes the observation data between the globally uniformly distributed ground stations and the satellites and solves the antenna phase center deviation parameters through network estimation [70].

#### 5.3.5. Ranging Noise

The inter-satellite pseudo-range values $\rho_{ij}$ and $\rho_{ji}$ are extracted from the pseudo-code tracking loop of the satellite receiving device. The precision of pseudo-range measurements is related to the thermal noise error and dynamic stress error of the tracking loop. The 1σ tracking error of the pseudo-code loop can be expressed as follows:

(46) $$\sigma_{DLL}=\sigma_{tDLL}+\frac{\theta_{e}}{3}$$

where $\sigma_{tDLL}$ is the tracking error caused by thermal noise, and $\theta_{e}$ is the dynamic stress error caused by inter-satellite relative movement. $\theta_{e}$ can be expressed in the following form as follows:

(47) $$\theta_{e}=\frac{1}{\omega_{n}^{N}}\frac{d^{N}D(t)}{dt^{N}}$$

where N is the order of the loop, and $\omega_{n}$ is the natural frequency in Hz. During actual tracking, carrier-aided code tracking technology is often used, which can largely eliminate most of the dynamic stress errors. Therefore, dynamic stress errors can be neglected, and the ranging error is primarily caused by thermal noise. $\sigma_{tDLL}$ can be expressed as follows:

(48) $$\sigma_{tDLL}=\frac{1}{f_{code}}\sqrt{\frac{2d^{2}B_{n}}{C/N_{0}}[2(1-d)+\frac{4d}{T_{coh}C/N_{0}}]}$$

where $f_{code}$ is the pseudo-code rate in Hz, $B_{n}$ is the equivalent noise band of the code loop in Hz, d is the correlation interval, and $T_{coh}$ is the coherent integration time. When the inter-satellite ranging system uses the CCFD scheme, compared to the traditional TDHD scheme, the measurement time can be doubled, and the system can reduce the equivalent noise band $B_{n}$, achieving higher-ranging accuracy by sacrificing convergence speed. Therefore, it can be theoretically verified that using the CCFD scheme can effectively reduce the pseudo-code ranging noise.

## 6. Experimental Verification and Analysis

To verify the feasibility of the CCFD scheme in the BDS-3 ISL scenario and analyze the ranging performance in CCFD, this section will conduct experimental simulations and analysis, mainly including the following four parts: (1) building the BDS-3 ISL simulation scenario; (2) analyzing the topological characteristics of the BDS-3 ISL, including inter-satellite visibility, inter-satellite distance, and its rate of change; (3) analyzing link budget to verify the feasibility of the CCFD scheme; (4) building a digital simulation system for two-way inter-satellite ranging, and analyzing the inter-satellite ranging performance in CCFD.

### 6.1. Simulation Scenario Construction

This section utilizes STK software to construct a BDS-3 ISL scenario. The orbital data for the simulation are sourced from the BeiDou satellite TLE orbital files available on the Celestrak website (https://celestrak.org/NORAD/elements/ (accessed on 1 November 2024)). The total simulation time range spans from UTCG 27 November 2024 04:00:00 to UTCG 1 December 2024 04:00:00.

The experiment selects MEO-19 as the first satellite in the first orbital plane of the MEO constellation (MEO_11_). At a given moment, when MEO_11_ passes the Right Ascension of the Ascending Node (RAAN), the satellite in the second orbital plane that reaches RAAN after 1/24 of the orbital period is defined as MEO_21_. Similarly, after another 1/24 period, the satellite in the third orbital plane reaching RAAN is defined as MEO_31_. This sequential logic is applied to assign identifiers to all satellites in the MEO constellation. The GEO and IGSO satellites are sequentially numbered from GEO_1_ to GEO_3_ and IGSO_1_ to IGSO_3_, respectively, in ascending order of their RAAN. The corresponding parameters for the BDS-3 are provided in Table 6.

**Figure 7:**
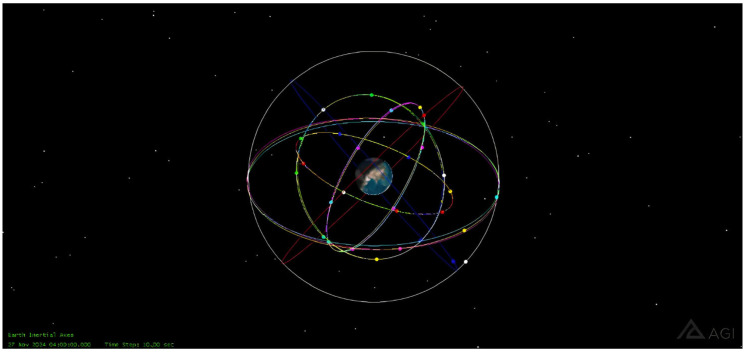
3D simulation scenarios of the BDS-3 constellation.

**Figure 8:**
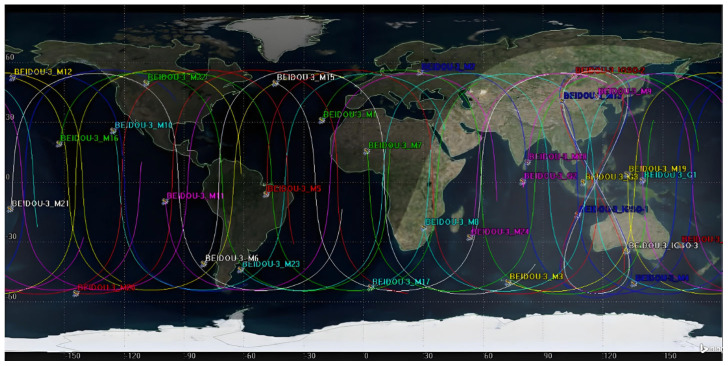
2D simulation scenarios of the BDS-3 constellation.

Figure 7 and Figure 8 display the 3D and 2D simulation scenarios of the BDS-3 constellation constructed based on STK software, respectively. The subsequent inter-satellite topology characterization and link budget analysis will be conducted within this established simulation scenario.

### 6.2. Analysis of Inter-Satellite Topology Characterization

**Figure 9:**
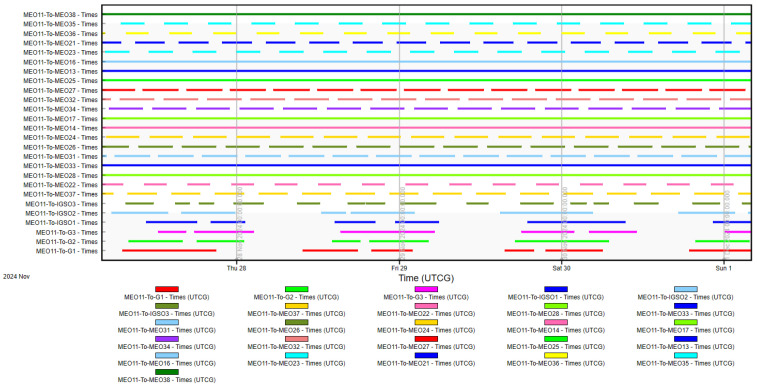
Visibility analysis of satellite MEO_11_ and other satellites in the BDS-3 constellation.

Based on the ISL dynamic constraint model established in Section 2, and taking into account both Earth occlusion and antenna beam scanning limitations, an analysis of inter-satellite visibility was conducted. Figure 9 presents the visibility analysis results for satellite MEO_11_ with all other satellites in the BDS-3 constellation.

**Figure 10:**
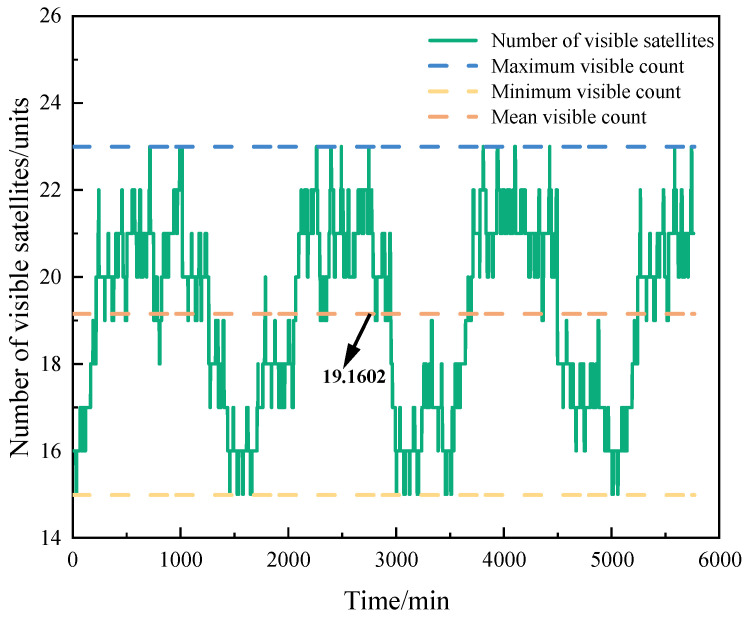
Variation in the number of visible satellites for satellite MEO_11_.

During the simulation period, satellite MEO_11_ maintained continuous visibility with 8 satellites, permanent invisibility with 3 satellites, and intermittent visibility with 18 satellites. Figure 10 illustrates the variation in the number of visible satellites for MEO_11_ throughout the simulation period. As shown in the figure, the maximum number of simultaneously visible satellites for MEO_11_ was 23, the minimum was 15, and the average stood at 19.1602.

**Figure 11:**
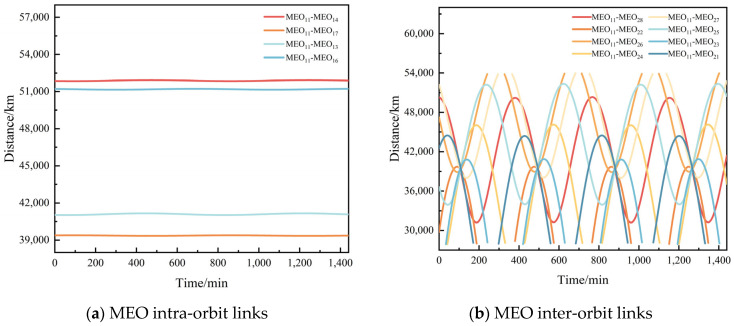

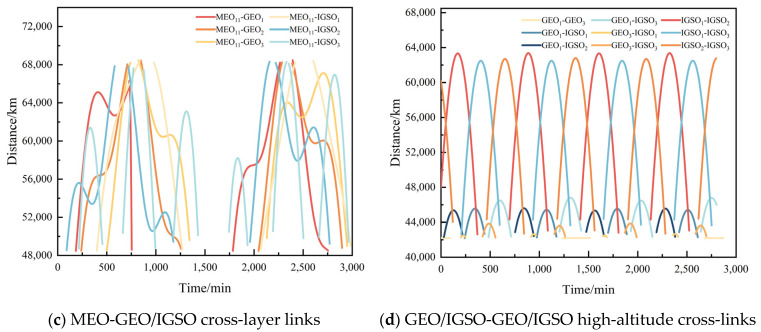
BDS-3 constellation inter-satellite distance characteristics.

The BDS-3 constellation features four distinct types of ISLs: MEO intra-orbit links (same orbital plane), MEO inter-orbit links (same altitude but different planes), MEO-GEO/IGSO cross-layer links, and GEO/IGSO-GEO/IGSO high-altitude crosslinks. After completing the inter-satellite visibility analysis, we further examined the inter-satellite distances and their rates of change. Figure 11 shows the inter-satellite distance characteristics for the four types of links in the BDS-3 constellation.

**Figure 12:**
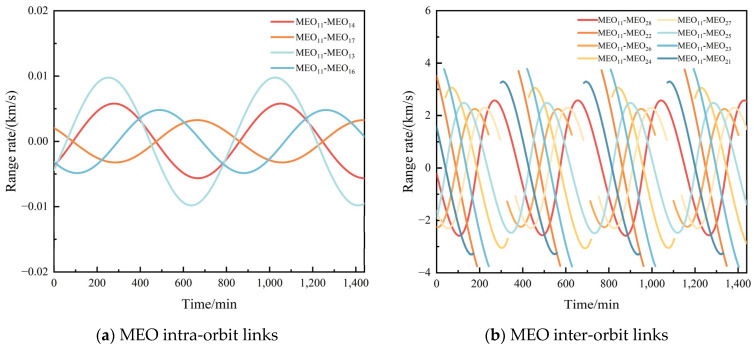

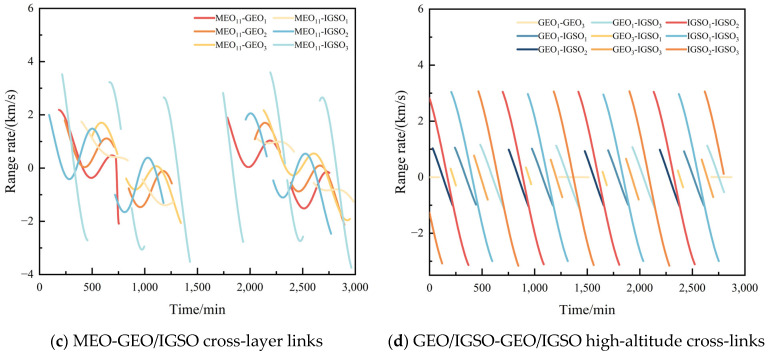
BDS-3 constellation inter-satellite range rate characteristics.

Figure 11 presents the inter-satellite distance characteristics of the BDS-3 constellation. Subfigure (a) shows the distances between satellite MEO_11_ and its co-orbital satellites, which remain essentially constant with a maximum distance of approximately 51,920 km and a minimum distance of about 39,342 km. Subfigure (b) displays the distances between MEO_11_ and satellites in the second orbital plane, ranging from a minimum of 27,909 km to a maximum of 53,948 km. Subfigure (c) illustrates the distances between MEO_11_ and high-altitude GEO/IGSO satellites, with maximum and minimum distances of about 68,609 km and 48,301 km, respectively. Finally, subfigure (d) demonstrates the distance characteristics between high-altitude GEO/IGSO satellites themselves, varying between approximately 42,164 km and 63,386 km. Figure 12 illustrates the characteristics of inter-satellite range rates for the four types of links.

Figure 12 presents the analysis of inter-satellite range rate characteristics for the BDS-3 constellation. Subfigure (a) shows the range rate between satellite MEO_11_ and its co-orbital satellites, demonstrating minimal variation with a maximum value of only about 0.001 km/s. Subfigure (b) displays the range rate between MEO_11_ and satellites in the second orbital plane, reaching a maximum of approximately 3.781 km/s. Subfigure (c) illustrates the range rate between MEO_11_ and high-altitude GEO/IGSO satellites, peaking at around 3.803 km/s. Finally, subfigure (d) depicts the range rate characteristics between high-altitude GEO/IGSO satellites themselves, with a maximum value of about 3.179 km/s.

Through the analysis of inter-satellite distances and range rates in the BDS-3 constellation, it is evident that the maximum inter-satellite distance reaches approximately 68,600 km, while the minimum is about 27,900 km—a difference exceeding 40,000 km. Additionally, the maximum range rate reaches approximately 3.8 km/s, fully demonstrating the high dynamicity and complex variability of inter-satellite motion in the BDS-3 system.

### 6.3. Analysis of Inter-Satellite Link Budget

Based on the BDS-3 topological characteristics, we proceed to analyze the link budget. Table 7 presents the key parameter values for the BDS-3 ISL link budget [2,71,72].

The system noise temperature $T_{s}$, noise power spectral density $N_{0}$, noise power N, required normalized signal-to-noise ratio ${\left(E_{b}/N_{0}\right)}_{req}$, and required carrier-to-noise ratio ${\left(C/N_{0}\right)}_{req}$ can be calculated using Equations (15), (16), (18) and (19). The computed parameter values are presented in Table 8.

**Figure 13:**
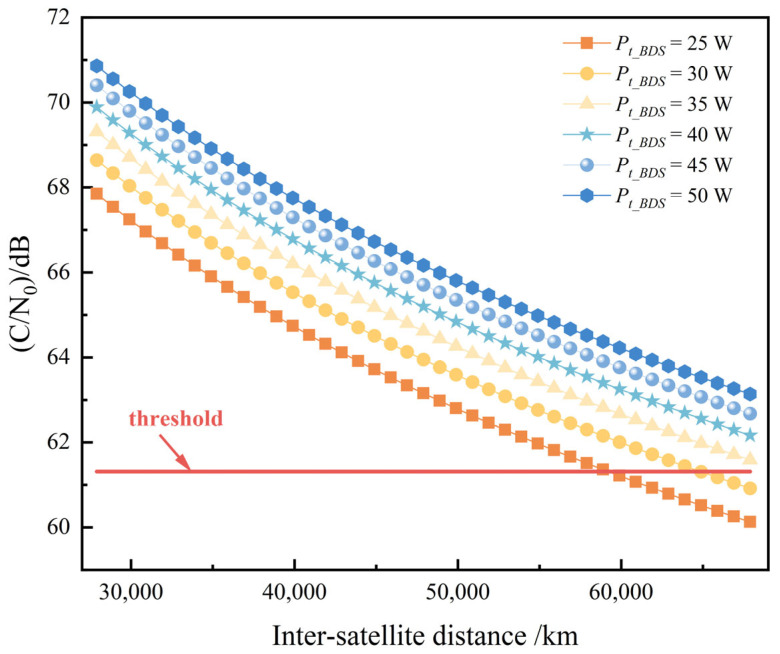
Carrier-to-noise ratio at the receiver in the case of transmission power variations.

To meet the system’s BER requirements while minimizing the demand for CCFD self-interference cancellation, the transmission power for BeiDou ISL signals was analyzed to determine the optimal power level. This experiment evaluated a transmission power sequence of [25 W, 30 W, 35 W, 40 W, 45 W, 50 W]. Figure 13 illustrates the carrier-to-noise ratio at the receiver in the case of transmission power variations.

The threshold value is set as the required carrier-to-noise ratio (${\left(C/N_{0}\right)}_{req}$) calculated in Table 8, i.e., 61.31 dBHz. It can be seen that in the whole process of ISL measurement, to satisfy the received carrier-to-noise ratio is always greater than the required carrier-to-noise ratio, the transmit power of the signal is selected as the most appropriate 35 W, and the range of the satellite received carrier-to-noise ratio in this case is 61.6 dBHz~69.3 dBHz. When the link margin is 0, the minimum transmit power can be derived as 33.5223 W using Equation (21). Theoretically, any transmit power exceeding this value would satisfy the requirements for inter-satellite measurement and communication. In this study, a transmit power of 35 W is selected, resulting in a link margin of approximately 0.187 dB, which meets practical application scenarios.

If the CCFD technology is introduced into the BDS-3 ISL communication and measurement system, it is necessary to ensure that the self-interference signal received at the satellite receiver can be suppressed below the receiver’s noise floor, thereby avoiding any impact on normal inter-satellite communication and measurement. The self-interference signal is transmitted through the antenna and directly received by the receiving antenna. By comprehensively considering factors such as transmission circuit loss and polarization loss, the required self-interference suppression amount for the system can be calculated as follows:

(49) $$\left[C]=[P_{t\_BDS}]+[G_{t\_BDS}]-[L_{S\_BDS}]-[L_{P\_BDS}]-[N\right]$$

where C is the required self-interference suppression amount. By incorporating the results calculated earlier, the system’s self-interference suppression amount can be determined to be 174.6 dB. Section 2 of the paper has indicated that the current level of self-interference suppression can reach 180 dB; thus, introducing the CCFD technology into the BDS-3 ISL scenario is feasible.

### 6.4. Analysis of Inter-Satellite Ranging Accuracy

This section analyzed the accuracy of inter-satellite pseudo-code ranging. First, the experimental simulation condition for pseudo-code ranging is provided, with specific parameter values shown in Table 9. Due to the rapid variation in inter-satellite distances and the significant Doppler shift between satellites, directly acquiring and tracking received signals is challenging. Therefore, navigation ephemeris data are often utilized to assist in practical inter-satellite link measurements. Since the navigation ephemeris can provide satellite positional accuracy at the meter level and velocity calculation precision of 0.1 mm/s, for an inter-satellite carrier frequency of 23 GHz and a code rate of 10.23 MHz, the corresponding delay and Doppler uncertainty regions are confined within half a code chip and 1 Hz, respectively. This allows the system to bypass the acquisition phase and proceed directly to the tracking phase. In this experiment, the delay and Doppler uncertainty regions are set to 0.5 code chips and 1 Hz, respectively, meeting the practical requirements of inter-satellite measurements.

**Figure 14:**
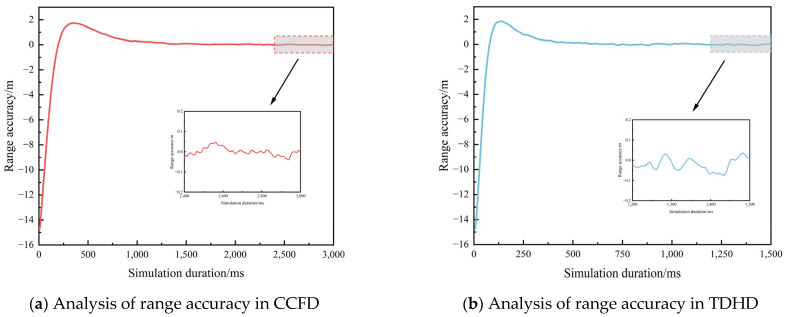
Comparison of ranging accuracy between CCFD and TDHD modes at a carrier-to-noise ratio of 61.6 dBHz.

**Figure 15:**
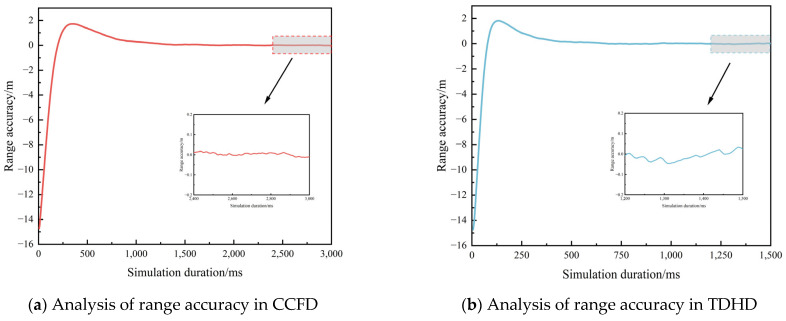
Comparison of ranging accuracy between CCFD and TDHD modes at a carrier-to-noise ratio of 69.3 dBHz.

To evaluate the performance enhancement of pseudo-code ranging in CCFD, this experiment developed a MATLAB-based digital simulation platform for inter-satellite measurement. The tracking subsystem implemented a carrier-aided code loop architecture to compensate for dynamic stress effects, featuring a non-coherent early-late power discriminator for code tracking and a dot product phase detector for carrier recovery. Considering the brief link establishment window characteristic of BDS-3 ISLs and the stringent temporal requirements for signal ranging, the experiment configured 1.5 s and 3 s as the ranging time thresholds for TDHD and CCFD systems, respectively. The reason for selecting 3 s as the measurement duration in CCFD is that the regime supports uninterrupted measurements in each time slot, so the total length of the time slot can be selected as the measurement duration. The experimental outcomes are presented in Figure 14 and Figure 15, demonstrating the comparative performance between operational modes.

According to the experimental simulation conditions, under the CCFD and TDHD schemes, selecting code loop equivalent noise bandwidths $B_{n}$ of 2 Hz and 5 Hz, respectively, can achieve better ranging accuracy and convergence performance. Figure 14 and Figure 15 present the simulation results of pseudo-code ranging accuracy under the two schemes at the maximum $C/N_{0}$ of 69.3 dBHz and the minimum $C/N_{0}$ of 61.6 dBHz, respectively. Analysis of the results indicates that the signal can achieve rapid convergence and stabilization within a short time under the simulated conditions. The last 20% of the time segment was selected for evaluating the ranging accuracy, and the results are summarized in Table 10.

Analysis of the experimental results reveals that at a $C/N_{0}$ of 61.6 dBHz, the 1σ ranging accuracy is 1.9 cm under the CCFD scheme and 3.9 cm under the TDHD scheme. Compared to the conventional TDHD scheme, the CCFD-based pseudo-code ranging accuracy improves by 51.3%. At a $C/N_{0}$ of 69.3 dBHz, the 1σ ranging accuracy is 0.8 cm under the CCFD scheme and 2.2 cm under the TDHD scheme. Compared to the conventional TDHD scheme, the CCFD-based pseudo-code ranging accuracy improves by 66.8%.

In summary, the analysis demonstrates that introducing CCFD technology into the BeiDou inter-satellite link system can effectively enhance pseudo-code ranging accuracy. This approach serves as an effective measure to meet future demands for higher-precision orbit determination and time synchronization.

## 7. Discussion

Theoretical analysis and experimental simulations confirm that the CCFD scheme can significantly enhance inter-satellite ranging accuracy, with its technical advantages manifested in two key aspects: Firstly, regarding observation precision, the CCFD scheme doubles the inter-satellite measurement duration, allowing the system to achieve narrower code loop equivalent noise bandwidth at the cost of moderately reduced loop convergence speed, thereby markedly improving pseudo-range observation accuracy (with improvement exceeding 50%). Secondly, in terms of timestamp reckoning, the CCFD system can effectively eliminate the “spatial reckoning” errors inherent in the conventional TDHD system caused by the difference between transmission and reckoning timestamps. After ephemeris and almanac corrections, residual errors can be maintained at negligible levels.

As a critical metric for BDS-3 ISL construction, ranging accuracy directly impacts system performance. Given that both orbit determination and clock offset calibration rely on pseudo-range observations, accuracy improvement is essential for achieving sub-meter precise orbit determination and nanosecond-level time synchronization, which will significantly enhance the navigation system’s service quality and constellation autonomy.

It is noteworthy that CCFD technology has applications extending far beyond BDS-3 ISLs. It demonstrates considerable potential in large-scale heterogeneous constellation networking, satellite formation flying, space station TT&C, and new technology demonstration satellites. While this study focuses on optimizing pseudo-code ranging methods, CCFD’s unique long-duration measurement capability creates opportunities for implementing higher-accuracy techniques (such as carrier-phase-smoothed pseudo-range and pure carrier-phase ranging), which will constitute our primary research direction. Furthermore, CCFD implementation not only enhances measurement performance but also improves inter-satellite data transmission efficiency and routing reliability; these derivative advantages warrant further investigation.

## 8. Conclusions

To address the requirement of high-precision ranging/orbit determination and high-capacity data transmission in BDS-3 ISLs, this study innovatively proposes a novel two-way measurement method based on a CCFD system. This method achieves significant ranging accuracy improvement through simultaneous same-frequency transceiving. The main work of this paper is as follows:

1. Analyzing the self-interference performance of the CCFD system from three aspects: spatial, RF, and digital domains, and evaluating the approximate total self-interference suppression in ISL scenarios;
2. Based on the BDS-3 navigation constellation configuration, constructing a dynamic link constraint model from two aspects of satellite visibility and antenna directivity;
3. Analyzing the link budget of BDS-3 ISL in the whole process, and giving the calculation methods of signal quality and link margin;
4. Providing the fundamental principle, mathematical model, and key error analysis of the inter-satellite two-way measurement method in CCFD, with particular emphasis on the theoretical derivation and analysis of inter-satellite timestamp reckoning accuracy;
5. Through experimental validation and analysis, establishing the BDS-3 ISL scenario using STK software to analyze inter-satellite topology characteristics. Based on the link budget results, verifying the feasibility of the CCFD system and demonstrating that its pseudo-code ranging accuracy achieves over 50% improvement compared with traditional systems, indicating promising application prospects.

This study systematically demonstrates the application value of the CCFD system in BDS-3 ISLs through theoretical analysis, algorithm design, and experimental verification. The continuous innovation of CCFD technology is expected to advance satellite navigation systems toward higher precision and greater autonomy.

## Figures and Tables

**Table 1:** Indicators of the operating characteristics of GPS satellites.

Satellite Type	Launch Time	Number	Operating Frequency	Duplex Mode
GPS-IIR	1997–2004	6	UHF	Half-duplex
GPS-IIR-M	2005–2009	7	UHF	Half-duplex
GPS-IIF	2010–2016	12	UHF	Half-duplex
GPS-III	2018–present	6	UHF, Ka, V	Half-duplex

**Table 2:** Status of spatial-domain self-interference suppression for array antennas.

Reference	Year	Central Frequency/GHz	Bandwidth/MHz	Suppression Method	Best Suppression Effect/dB
[40]	2020	19.7~21	\	Orthogonal polarization	45
[41]	2020	21.5	\	Parasitic patches	52.7
[42]	2021	28	80	Cross-polarization	57
[43]	2021	28	500	Linear patch	100
[44]	2022	24.125	500	Feed structure decoupling	70
[45]	2022	26~27	100	Orthogonal polarization	77
[46]	2023	26.5	200	Electromagnetic bandgap structures	109.1
[47]	2024	17.7~21.4	\	Matching network design	67

**Table 3:** Status of RF-domain self-interference suppression.

Reference	Year	Central Frequency /GHz	Bandwidth /MHz	Suppression Method	Best Suppression Effect/dB
[51]	2015	25	10	Mach–Zehnder modulator design	27
[52]	2019	28	1024	Rat-Race coupler design	40
[53]	2020	28	\	Hybrid transformer design	28.4
[54]	2021	28	400	80-tap RF cancellation	30
[45]	2022	26~27	100	16-tap channel reconstruction	37

**Table 4:** Status of digital-domain self-interference suppression.

Reference	Year	Bandwidth/MHz	Suppression Method	Best Suppression Effect/dB
[59]	2021	10	IVSSLMS adaptive filtering algorithm	48
[60]	2022	20	Digital cancellation of PA nonlinearity and IQ imbalance	72
[61]	2023	100	Self-interference channel reconstruction	37.78
[62]	2023	750	Bi-LSTM-based self-interference channel estimation	47.17
[63]	2024	2000	Deep learning-based self-interference signal	60.62

**Table 5:** Calculation results of maximum communication distance and Earth obstruction angle.

Parameter	Description	Value	Unit
$L_{\max(MEO-MEO)}$	Maximum distance for MEO-MEO links	55,897.12	km
$L_{\max(MEO-GEO/IGSO)}$	Maximum distance for MEO-GEO/IGSO links	70,477.33	km
$\alpha_{MEO}$	Earth occlusion angle for MEO satellites	14.79	°
$\alpha_{GEO/IGSO}$	Earth occlusion angle for GEO/IGSO satellites	9.84	°

**Table 6:** The corresponding parameters for the BDS-3.

Satellite ID	Satellite Type	PRN	Launch Time
MEO_11_	MEO-19	C41	16 December 2019
MEO_12_	MEO-13	C32	19 September 2018
MEO_13_	MEO-02	C20	5 November 2017
MEO_14_	MEO-01	C19	5 November 2017
MEO_15_	MEO-04	C22	12 February 2018
MEO_16_	MEO-03	C21	12 February 2018
MEO_17_	MEO-20	C42	16 December 2019
MEO_18_	MEO-14	C33	19 September 2018
MEO_21_	MEO-06	C24	29 July 2018
MEO_22_	MEO-11	C25	24 August 2018
MEO_23_	MEO-05	C23	29 July 2018
MEO_24_	MEO-18	C37	18 November 2018
MEO_25_	MEO-24	C46	22 September 2019
MEO_26_	MEO-17	C36	18 November 2018
MEO_27_	MEO-23	C45	22 September 2019
MEO_28_	MEO-12	C26	24 August 2018
MEO_31_	MEO-16	C35	15 October 2018
MEO_32_	MEO-22	C44	23 November 2019
MEO_33_	MEO-15	C34	15 October 2018
MEO_34_	MEO-21	C43	23 November 2019
MEO_35_	MEO-08	C28	11 January 2018
MEO_36_	MEO-07	C27	11 January 2018
MEO_37_	MEO-10	C30	29 March 2018
MEO_38_	MEO-09	C29	29 March 2018
GEO_1_	GEO-02	C60	29 March 2020
GEO_2_	GEO-03	C61	23 June 2020
GEO_3_	GEO-01	C59	1 November 2018
IGSO_1_	IGSO-01	C38	20 April 2019
IGSO_2_	IGSO-02	C39	24 June 2019
IGSO_3_	IGSO-03	C40	5 November 2019

**Table 7:** The key parameter values for the BDS-3 ISL link budget.

Parameter	Value	Unit
$f_{BDS\_ISL}$	23	GHz
$G_{t\_BDS}$	35	dBi
$G_{r\_BDS}$	35	dBi
$L_{S\_BDS}$	2	dB
$L_{N\_BDS}$	2	dB
$L_{P\_BDS}$	1	dB
$L_{D\_BDS}$	0.3	dB
$B_{f}$	20.46	MHz
F	4	dB
$T_{a}$	100	K
$T_{0}$	290	K
$R_{b\_ISL}$	100	kbps
$L_{M}$	3	dB
$P_{b}$	10^−7^	\

**Table 8:** The computed parameter values for the BDS-3 ISL link budget.

Parameter	Value	Unit
$T_{s}$	964.51	K
$N_{0}$	−198.76	dBW/Hz
N	−125.65	dBW
${\left(E_{b}/N_{0}\right)}_{req}$	11.31	dB
${\left(C/N_{0}\right)}_{req}$	61.31	dBHz

**Table 9:** The key parameter values for the BDS-3 ISL pseudo-code ranging.

Parameter	Description	Value	Unit
$f_{code}$	Pseudo-code rate	10.23	MHz
$f_{BDS\_ISL}$	Carrier frequency	23	GHz
$f_{d}$	Doppler shift uncertainty region	1	Hz
$\tau_{d}$	Code delay uncertainty region	0.5	chip
$B_{L}$	Noise band of the carrier loop	25	Hz
d	Correlation interval	0.5	\
$T_{coh}$	Coherent integration time	2	ms
$T_{non\_coh}$	Non-coherent integration times	3	\
$G_{carrier}$	Carrier loop gain	0.01	\
$G_{code}$	Code loop gain	0.01	\
$C/N_{0}$	Carrier-to-noise ratio	61.6~69.3	dBHz

**Table 10:** Comparison of pseudo-code ranging accuracy based on CCFD and TDHD schemes.

Ranging System	*C*/*N*_0_/dBHz	1σ Ranging Accuracy/cm
CCFD	61.6	1.9
	69.3	0.8
TDHD	61.6	3.9
	69.3	2.2

## Data Availability

Some of the data are publicly available at https://celestrak.org/NORAD/elements/ (accessed on 1 November 2024) and http://www.beidou.gov.cn/ (accessed on 14 March 2025). The data presented in this study are available on request from the corresponding author.

## Abbreviations

The following abbreviations are used in this manuscript:

CCFD	Co-frequency Co-time Full Duplex
ISL	Inter-satellite link
GNSS	Global Navigation Satellite System
GPS	Global Positioning System
URE	User range error
ESA	European Space Agency
TDMA	Time-division multiple access
FTDMA	Frequency-hopping time-division multiple access
OQL	Optical quantum links
BDS-3	BeiDou-3 Global Navigation Satellite System
PNT	Positioning, Navigation, and Timing
MEO	Medium Earth Orbit
GEO	Geostationary Earth Orbit
TDHD	Time-division half-duplex
SOI	Signal of Interest
SIS	Self-Interference Signal
RF	Radio frequency
IF	Intermediate frequency
IGSO	Inclined Geosynchronous Orbit
SNR	Signal-to-noise ratio
BER	Bit Error Rate
BDT	BeiDou time
PCO	Phase center offset
ECI	Earth-Centered Inertial
NFO	Nominal frequency offset
RAAN	Right Ascension of the Ascending Node
